# Mining nucleic acid “omics” to boost liquid biopsy in cancer

**DOI:** 10.1016/j.xcrm.2024.101736

**Published:** 2024-09-17

**Authors:** Ann Tivey, Rebecca J. Lee, Alexandra Clipson, Steven M. Hill, Paul Lorigan, Dominic G. Rothwell, Caroline Dive, Florent Mouliere

**Affiliations:** 1 Cancer Research UK National Biomarker Centre, University of Manchester, Manchester, UK; 2 Division of Cancer Sciences, University of Manchester, Manchester, UK

## Abstract

Treatments for cancer patients are becoming increasingly complex, and there is a growing desire from clinicians and patients for biomarkers that can account for this complexity to support informed decisions about clinical care. To achieve precision medicine, the new generation of biomarkers must reflect the spatial and temporal heterogeneity of cancer biology both between patients and within an individual patient. Mining the different layers of 'omics in a multi-modal way from a minimally invasive, easily repeatable, liquid biopsy has increasing potential in a range of clinical applications, and for improving our understanding of treatment response and resistance. Here, we detail the recent developments and methods allowing exploration of genomic, epigenomic, transcriptomic, and fragmentomic layers of 'omics from liquid biopsy, and their integration in a range of applications. We also consider the specific challenges that are posed by the clinical implementation of multi-omic liquid biopsies.

## Graphical abstract

**Figure:**
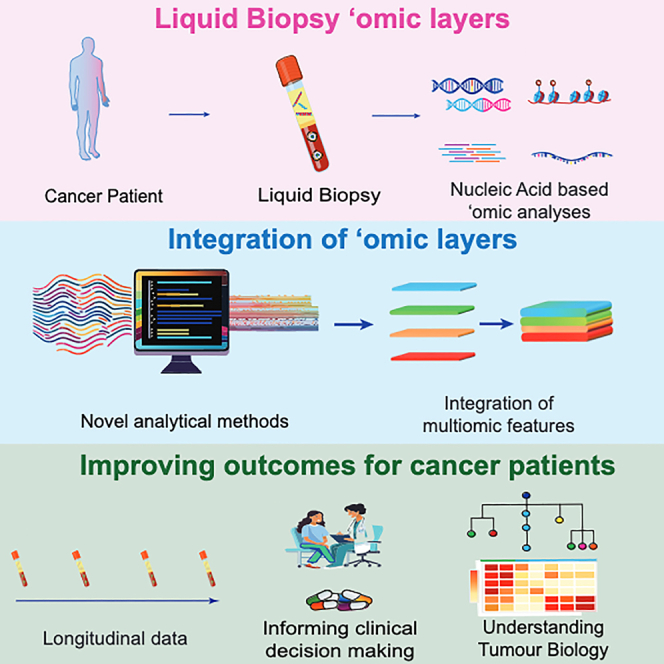

## Teaser

Mining the different “omics” layer from a liquid biopsy has potential application in a range of clinical settings and could improve understanding of treatment response and resistance. In this review, Tivey et al. detail recent developments allowing exploration of genomic, epigenomic, fragmentomic, and transcriptomic layers from liquid biopsy.

## Introduction

The recent and rapid expansion of targeted and immunotherapy options across multiple cancer types has been associated with improved patient outcomes. In parallel, there is growing interest in tools that can guide treatment decisions in an increasingly complex therapeutic landscape, enabling physicians to tailor aspects of treatment such as choice of drug and schedule to an individual patient and the patient’s cancer. Biomarkers that provide prognostic and/or predictive information are essential to guide such decisions. Understanding tumor biology in an individual patient is often based on tissue biopsy samples which can be analyzed for a range of genetic, transcriptomic, epigenomic, proteomic, and metabolomic biomarkers. There are limitations of tissue-based biomarkers including a failure to capture heterogeneity when only small biopsies are used and the challenges of longitudinal tissue sampling while on treatment. “Liquid biopsies,” where the biomarker can be present in a more readily and repeatedly accessible biological fluid, overcome some of these barriers.

**Figure 1:**
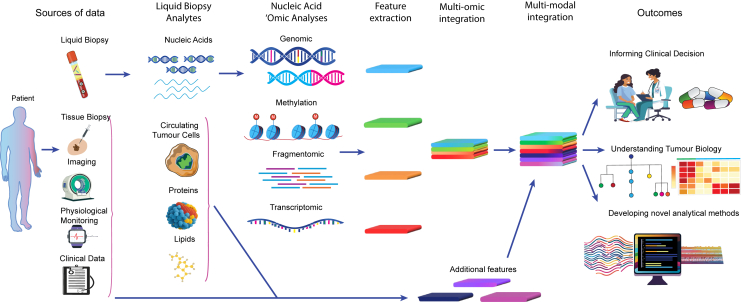
The potential of multi-omic liquid analytes

A wide range of liquid biopsy analytes are evaluable, including cell-free DNA (cfDNA), cell-free RNA (cfRNA), circulating tumor cells (CTCs), extracellular vesicles, proteins, lipids, tumor educated platelets, mitochondria, and neutrophil extracellular traps^1^^,^^2^^,^^3^^,^^4^^,^^5^ (Figure 1). The biogenesis and potential application of these analytes differ, with for example CTCs being implicated in development of metastases, thus being both a potential biomarker and a therapeutic target.^6^^,^^7^ Liquid biopsy research has focused on blood-based biomarkers, but there is also growing interest in alternative bio-fluids such as urine, cerebrospinal fluid (CSF), saliva, and tears.^8^ Nucleic acid-based liquid biopsies in particular are able to generate large amounts of 'omic data through next-generation sequencing (NGS) approaches.^9^ The distribution of nucleic acids in the different blood analytes can be variable and remains to be fully characterized; for example, there is conflicting evidence regarding the content and nature of nucleic acids in extracellular vesicles.^10^^,^^11^^,^^12^ This review will focus on evaluating the challenges and opportunities multi-omic analyses of cell-free nucleic acids (cfNAs) from plasma can offer for improving our understanding and management of cancer.

cfNAs are fragments of DNA (cfDNA) or RNA (cfRNA) existing outside of cells in a bio-fluid. cfNA can be found in healthy individuals and individual with cancers or other pathologies.^13^^,^^14^^,^^15^^,^^16^ Circulating tumor DNA/RNA (ctDNA/RNA) refers to cfNAs derived from tumor cells. The majority of cfNAs are derived from hematopoietic cells; this is an important consideration when designing experiments and developing assays for clinical use, as assay sensitivity will be impacted by the tumor fraction and intended clinical application.^13^^,^^14^^,^^17^^,^^18^

## Analyzing “omics” layers from liquid biopsy

### Genomic analysis of cfDNA

There are an increasing number of approaches for studying cfNAs (Table 1). Initial focus was on analyzing the genomic content of ctDNA, including mutations at specific genes using PCR and sequencing-based approaches. Digital PCR (dPCR) and digital droplet PCR (ddPCR) are sensitive and fast techniques for detection and quantification of mutant DNA copies down to variant allele frequencies (VAFs) as low as 0.01%.^19^^,^^20^^,^^21^ This sensitivity and simplicity of use led to studies using ddPCR to monitor minimal residual disease (MRD), for example, the early detection of melanoma relapse post-surgery.^20^^,^^22^ However, ddPCR cannot inform on unknown variants, intra-tumor heterogeneity, or tumor evolution.

**Table 1:** Comparison of different analytical approaches for study of cfDNA

	Coverage	Output	Turnaround time from taking sample	Suggested minimum cfNA input	Relative complexity of analysis and clinical interpretation	Relative cost £-££££	Fragment information preserved?
**Genomic analyses**							

ddPCR	1 mutation hotspot/assay	VAF (quantitative)	2–5 days	0.1 ng/well	Low	£	No
Targeted NGS	Up to 1000 genes	Base pair sequence	1–2 weeks	10 ng	Medium	££	At selected regions only for hybrid capture-based methods
WES	All protein coding regions (∼1.5% genome)	Base pair sequence, copy-number changes	2 weeks	10 ng	Medium-High	£££	At exome only
WGS	Whole genome	Base pair sequence, copy-number changes	2–3 weeks	10 ng	High	££££	Yes

**Methylation analyses**							

Bisulfite conversion based sequencing	Up to whole methylome but loss of 84–96% DNA	Methylation status at base pair level.	2 weeks	10 ng, ideally more for WGBS	High	££-£££^a^	At regions covered
Methylation capture-based enrichment	Genome-wide methylation	Methylation status at fragment level (5-mC only)	2–3 weeks	1 ng	High	£££	Only for enriched methylated regions
Enzyme-based methylation (e.g., EM-seq, TAPs)	Up to whole methylome	Methylation status at base pair level.	2–3 weeks	10 ng	High	£££-££££^a^	Yes

**Combined genomic and methylation analyses**							

5/6 letter sequencing (Duet)	Up to whole methylome/whole genome	5-mC/5-hMC status at base pair level. Preserves base pair sequence.	2–3 weeks	5 ng	High	£££- ££££^a^	Partially, dependent on read length
Native DNA sequencing e.g., nanopore	Up to whole methylome/whole genome	5-mC/5-hMC status at base pair level. Preserves base pair sequence. Sequences longer fragments	24–48 h	5 ng	High	£££-££££^a^	Yes

**Transcriptomic analyses**							

ddPCR	1 gene/assay	VAF (quantitative)	2–5 days	0.1 ng/well	Low	£	No
Targeted panel	Up to 1000 genes	Base pair sequence, fusions	1–2 weeks	10 ng	Medium	££	n/a
RNA-seq (whole exome/whole transcriptome)	All protein coding (exome)/transcribed regions	Base pair sequence, copy-number changes	2 weeks	10 ng	Medium-High	£££	n/a

VAF, variant allele frequency; ddPCR, digital droplet polymerase chain reaction; NGS, next-generation sequencing; WGS, whole-genome sequencing; WES, whole-exome sequencing.

a Cost varies dependent on sequencing coverage and depth.

Sequencing techniques provide information ranging from point mutations to copy-number alterations, and large structural changes (e.g., aneuploidy, whole-genome duplication, or structural variations).^23^ Whole-genome sequencing (WGS) enables *de novo* discovery of mutations implicated in tumorigenesis or resistance from within both coding and non-coding regions of the genome. Due to the genome size with increasing read depths, there is a significant cost associated with WGS at sufficiently high depth to accurately detect rare variants which is a disadvantage in the context of cfDNA where VAF can be minimal. The vast quantity of data generated by WGS also requires substantial processing power and storage. Lower-depth WGS can, however, still be very effective for identifying copy-number alternations (CNAs), as well as cfDNA biological features such as fragment length or ending.^24^

Whole-exome sequencing (WES) limits sequencing to the protein coding genome through hybridization enrichment and is more sensitive in detecting low VAF on a genome-wide scale than WGS.^25^ However, it is uninformative on non-coding regions such as enhancers which, when mutated, can still have significant biologic consequences. Targeted sequencing panels involve selective enrichment of a small number of genes/genomic regions (∼50–1,000) prior to sequencing and can achieve high read depths at a moderate cost.^26^ Deep targeted sequencing can increase sensitivity for selected variants present at low VAF.^27^ This is however limited by the choice of genes in a particular panel and therefore less suited for *de novo* discovery of novel mutations/resistance mechanisms, or for assessment of large structural changes.

For mutation-based analysis of cfDNA, it is important to distinguish true tumor-derived mutations from those arising from clonal hematopoiesis of indeterminate potential (CHIP).^28^^,^^29^ Ideally this is achieved through parallel sequencing of either tumor or white blood cell DNA although this impacts cost and practicality and in the case of the former is limited to calling mutations that are present in the initial tumor biopsy sample.

### cfDNA methylome profiling

The number of mutations in a cancer cell can be limited in some malignancies, whereas the number of altered methylated sites can be higher and occur early in cancer development, increasing the chances to detect tumor signal or infer more complex information from liquid biopsies with this additional layer of omics data.^30^

DNA methylation is the transfer of a methyl group onto a nucleotide, which occurs most commonly at a cytosine residue adjacent to a guanine residue (CpG site) to form 5-methylcytosine (5-mC).^31^^,^^32^ This epigenetic modification can regulate gene expression by affecting transcription factor binding and by influencing chromatin structure.^33^ Methylation patterns in healthy differentiated cells are generally stable and cell type specific.^13^^,^^34^ Global hypomethylation is a feature of many malignancies and can result in nuclear disorganization and loss of gene silencing of genes involved in cellular proliferation.^35^ Conversely, hypermethylation at specific sites (commonly within regions with high frequency of CpG sites) involving promoters and transcription factor binding sites (TFBSs) of key tumor suppressor genes may silence those genes.^32^

Methylation profiles therefore can be used to determine the tissue of origin of cfDNA, classify between cancer and non-cancer, and classify cancer subtypes.^13^^,^^14^^,^^36^^,^^37^^,^^38^ Methylation changes are often an early step in tumorigenesis and can be more numerous across the genome than genetic alterations Therefore sensitivity of methylation-based methods can be greater than that of mutation-based techniques, which may be particularly important in settings such as early cancer detection.^30^^,^^32^^,^^39^

Several techniques exist for studying methylation (in both cfDNA and other analytes) (Table 1). These include methods based on conversion of unmethylated cytosines to uracil, either by bisulfite treatment or by enzymatic methods, prior to PCR amplification and sequencing.^37^^,^^40^^,^^41^^,^^42^^,^^43^ An alternative approach is capture-based enrichment where methylated cytosine binding antibodies or proteins enrich for methylated DNA fragments.^30^^,^^36^^,^^44^ One caveat of capture-based methylation workflows is that they produce a stronger signal for differentially hypermethylated regions than hypomethylated regions which may be important for determining cfDNA tissue of origin, or deconvoluting the cell of origin of cfDNA.^13^

Methods that could provide both methylation and genomic analyses from a single workflow would be attractive in the context of a multi-omic liquid biopsy study. A recently developed method uses enzymatic conversion methods but preserves accurate sequencing of the underlying nucleotide sequence, enabling genomic and methylation analyses from a single workflow, although there is limited data on its use on cfDNA.^45^ Oxford Nanopore sequencing negates the requirement for conversion or enrichment steps by directly sequencing the native molecule without PCR amplification based on changes in electrical signal as it passes through a nanopore. This technology can distinguish unmethylated cytosine from 5-mC and provide genomic and methylation data from a single sequencing run. This has been used successfully in cfDNA to identify cancer-specific methylation profiles although there are challenges in optimizing nanopore technology for cfDNA fragment size and lower inputs.^46^^,^^47^

### cfDNA fragmentomics

The described methods preserve to a varying extent the cfDNA structural features, which can be altered in cancer, offering potential for adding an additional “omic” layer to analyses of data from sequencing workflows. Fragmentomics refers to the concept of inferring biological information such as gene expression, cell of origin, and mechanism of cell death using the structural properties of cfDNA.^48^ There are differences between tumor- and non-tumor-derived cfDNA fragment size profiles.^49^^,^^50^ The majority of cfDNA originating from both healthy and cancer cells is released during apoptosis, although other sources include necrosis and active secretion by cancer cells.^51^^,^^52^ Where chromatin is tightly wrapped or “closed” as in hematopoietic cell apoptosis (source of the majority of cfDNA), cfDNA cleavage mainly occurs through cell death nuclease-induced cleavage within the 20 bp DNA linker region, while the 147 bp DNA wrapped around the histone is protected. Hence, fragmentation is non-random and generates the 167 bp peak seen in cfDNA from healthy control subjects.^53^ In cancer cells, there is often a greater degree of transcriptional activity (with associated global hypomethylation) and consequentially more open chromatin cleavage with more sites accessible for cleavage. This enriches the cfDNA profile for shorter fragments, with periodic 10 bp peaks related to the repeating structural unit of the DNA double helix.^49^^,^^50^^,^^54^ Moreover, recent works have highlighted the sharp presence of ultrashort cfDNA fragments (around 50 bp) using single-stranded DNA sequencing,^55^ and a large proportion of tumor-derived cfDNA fragments >300 bp using Nanopore sequencing.^56^

Fragmentomics approaches can be applied to sequencing data from either genomic or methylation-based studies offering the potential for integration of multiple omics layers from a single experiment in a multi-modal way. Examples of fragmentomic features include fragment lengths, fragment-end motifs, fragment-end positions, jaggedness, and fragment orientation (Table 2).^54^^,^^57^^,^^58^^,^^59^^,^^60^^,^^61^ Tumor-specific fragment properties can be integrated with genomic information to improve sensitivity of existing liquid biopsies. For example, the size profile can discriminate between tumor- and non-tumor-derived cfDNA and aid with filtering of CHIP variants.^54^^,^^62^^,^^63^^,^^64^ Furthermore, transcriptional activity can be inferred based on patterns of fragmentation around transcription start sites (TSSs) and TFBSs. These data can in turn be informative about cfDNA tissue of origin (both tumor- and non-tumor-derived cfDNA) and characterize different cancer subtypes with distinct transcriptional programming.^59^^,^^65^^,^^66^^,^^67^ A small number of studies have successfully used enzymatic-based methylation sequencing to obtain methylation and fragment length profiles which were integrated to develop cancer detection and tissue-of-origin classifiers.^37^^,^^68^^,^^69^ The performance of such approaches to infer the tissue of origin compared to methylation-based assays remains to be demonstrated. As transcriptional activity is a dynamic process that can change over time with associated epigenetic modification, a fragmentomic approach may be able to track changes in gene expression patterns longitudinally within a single patient, providing insight into both relative subclone prevalence and the development of non-genetic resistance mechanisms. An additional layer of information on gene expression could be provided by analysis of cfRNA.

**Table 2:** Selected studies utilizing different fragmentomics approaches

Study	Fragment feature	Application	Technology	Key findings and limitations
Mouliere et al. 2018^54^	Fragment size difference between cancer and healthy on a global level, 10 bp oscillations on subnucleosomal level	Cancer diagnosis (pan tumor)	Shallow WGS, WES, size selection enrichment	AUC 0.91–0.99 depending on cancer type. Low sequencing depth (0.4x). Sensitive at low MAFs (after size specific enrichment. Only late-stage cancers
Cristiano et al. 2019^61^	Fragment size difference between cancer and healthy on a regional level	Cancer diagnosis (pan-tumor)	Delfi (WGS)	AUC 0.94 (Sens 57–99%, Spec 98%) supervised model, non-age matched controls
Ulz et al. 2019^59^	Coverage at TFBS and TSS	Prostate cancer subtyping and early detection	WGS	High tumor fraction required
Snyder et al. 2016^65^	Fragment endpoints, coverage near TFBS and near TSS	Cell of origin	Windowed protection score – WPS (WGS inc single strand)	Single strand sequencing enriched shorter fragments. Small sample numbers, high sequencing depth required (∼100x).
Esfahani et al. 2022^70^	Fragment length diversity at promoter regions ‘promoter fragment entropy’, coverage at regions near TSS (“nucleosome depleted regions”)	Diagnosis and subtype classification (lung cancer, diffuse large B cell lymphoma)	EPIQ-Seq (Targeted sequencing, also used WGS/WES)	Composite model of PFE/NDR, Lung cancer from healthy AUC 0.91 (training), 0.83 (validation), NSCLC subtype AUC 0.9, DLBCL from healthy AUC 0.92 (training) AUC 0.96 (validation). Requires disease specific panels for EPIC-seq, less sensitive at early stage
De Sarkar et al. 2023^66^	Coverage at TFBS and TSS, nucleosome phasing (periodicity of nucleosome positions)	Prostate cancer phenotyping	Keraon, ctdPheno (WGS)	AUC 0.96 (90.4% sensitivity, 97.5% specificity) for phenotyping. Lower limit of 8% and 3% tumor fraction required. (ctDPheno/Keraon respectively)
Sun et al. 2019^57^	Strand orientation	HCC diagnosis and tissue of origin	Orientation-aware cfDNA fragmentation- OCF (WGS)	67% sensitivity, 93.8% specificity for HCC. Lower tissue fraction required than some approaches e.g., ∼5%. Based on known open chromatin regions with limited independent validation.
Jiang et al. 2020^58^	End-motif frequency	Cancer diagnosis (mainly HCC)	WGS, WGBS	AUC 0.86 for HCC. Accurate at 4% tumor fraction. Requires deep sequencing for accuracy. Limited independent validation.
Doebley et al. 2022^71^	TFBS coverage by fragment midpoint	Cancer detection (pan-tumor), breast cancer subtyping.	Griffin (WGS)	Ultra-low-pass WGS (0.1x). Cancer vs. non-cancer: AUC 0.89 for 0.1x coverage. AUC 0.92 for breast cancer subtyping. Mainly existing cohorts, limited independent validation.

WGS, whole-genome sequencing; WES, whole exome sequencing; WGBS, whole genome bisulfite sequencing; TSS, transcription start site; TFBS, transcription factor binding site; HCC, hepatocellular carcinoma; NSCLC, non-small cell lung cancer; VAF, variant allele frequency; PFE, promoter fragment entropy; NDR, nucleosome depleted regions; AUC, area under the curve.

### cfRNA analyses

cfRNA is a distinct analyte that can be isolated in parallel with cfDNA. Only a small fraction (∼2%) of extractable RNA is messenger RNA (mRNA) with non-coding, ribosomal, and mitochondrial RNA representing ∼95% of cfRNA molecules, although, by isolating extracellular vesicles, the relative fraction of mRNA can increase up to ∼20%.^18^^,^^72^^,^^73^ Although theoretically more abundant than cfDNA due to multiple copies of mRNA per cell, unbound RNA in the circulation is potentially more vulnerable to degradation by RNases.^18^ As with cfDNA, cfRNA is released by dying cells but in addition can be actively secreted with extracellular vesicles which can protect RNA from degradation.^74^ cfRNA may also be protected from RNases by binding to proteins (e.g., microRNAs bound to RNA-induced silencing complex proteins) or lipoproteins.^75^^,^^76^ The active secretion of RNA means that it may be theoretically more abundant than cfDNA in certain situations such as tumors that are smaller or with slower turnover where the quantity of cfDNA released in cell death is limited, although this has not to date been demonstrated.

Different blood preservation tubes and processing conditions can significantly impact RNA stability leading to challenges in reproducibility.^77^ There are also challenges relating to the lack of standardized approaches for normalization of cell-free mRNA and reference genes datasets which limit reproducibility of existing methods. Nevertheless, cfRNA represents a distinct analyte able to discriminate patients with cancer from non-cancer patients and those with precancerous lesions.^18^^,^^78^^,^^79^^,^^80^ One study applied whole-transcriptome sequencing to detect distinct gene signatures present in plasma from patients with lung and breast cancer that were absent from non-cancer controls.^18^ Repetitive, non-coding RNA sequences such as transposable elements may also be enriched in cfRNA of cancer patients, with characteristic cancer type-specific profiles, and may be another source of ‘omic information that could be integrated in cancer detection and tissue of origin classifiers.^81^ cfRNA could also present advantages compared to cfDNA for the detection of fusions as the large intronic regions that can have multiple different breakpoints are not present in the RNA transcript simplifying the design of targeted panels for specific fusions.^82^

Cell-free transcriptomic data have also been integrated with genomic and epigenomic data in patients with gastrointestinal cancers and healthy controls. Notably, the correlation between gene expression data and genomic/epigenomic data in plasma was lower than that in previously published datasets from tissue/cell-lines, likely as a consequence of the distinct cellular origins of cfDNA and cfRNA.^83^ The authors demonstrated that the different ‘omic layers were complementary when applied to detection of cancer-specific genetic mutations, with variants being detected with greater sensitivity in cfRNA than cfDNA.

**Figure 2:**
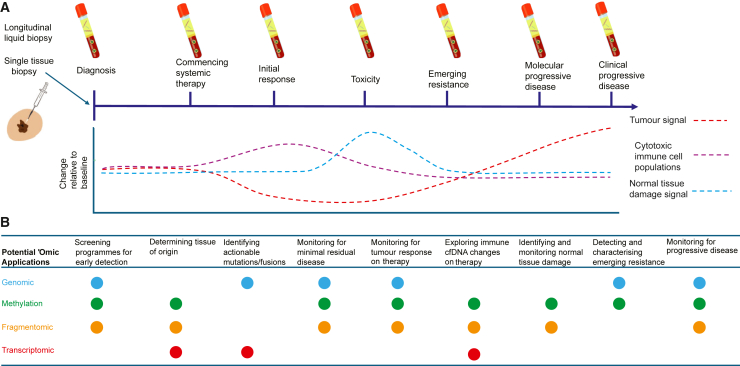
Applications of longitudinal multi-omic liquid biopsy (A and B) (A) Potential changes in cfDNA at different time points during cancer diagnosis and treatment including tumor-derived ctDNA (red), immune-cell-derived cfDNA (purple), and normal tissue-derived cfDNA during toxicity (green). (B) 'Omic applications that could be fulfilled by different 'omic layers at different time points.

Liquid biopsies have been studied in a range of clinical settings at different time points (Figure 2). We now consider specific examples where multi-omic analyses may be applied both to directly influence patient management and to improve understanding of tumor biology and therapeutic resistance.

## Circulating tumor NAs as a biomarker for clinical decision making

### Cancer detection and diagnosis

A challenge for developing liquid biopsy screening tests is the lower levels of cfNAs in earlier stage disease. The initial testing of the Galleri bisulfite methylation test in 4,077 patients (cancer, *n* = 2,823; non-cancer, *n* = 1,254) reported an overall sensitivity of 51.5%, but for stage I and II disease it was only 27.5% compared to 90.1% in stage IV.^84^ This was further validated in the SYMPLIFY study which detected a positive predictive value of 75.5% (95% confidence interval 70.5–80.1), negative predictive value of 97.6% (97.1–98.0), sensitivity of 66.3% (61.2–71.1), and specificity of 98.4% (98.1–98.8) of the Galleri test in patients presenting with symptoms suggestive of cancer.^85^^,^^86^ The SEPT9 methylation bisulfite-PCR-based assay is Food and Drug Administration (FDA) approved for colorectal cancer (CRC) screening, although meta-analysis has suggested in an asymptomatic population that it is inferior to existing fecal immunohistochemical assays and it has not yet been widely implemented outside of research studies.^87^^,^^88^ More recently, the ECLIPSE clinical trial (NCT04136002) examining a commercial cfDNA test incorporating methylation, genetic, and fragment features in 7,861 patients undergoing colonoscopy screening showed 83% sensitivity for CRC, 90% specificity for advanced neoplasia, and 13% sensitivity for advanced precancerous lesions.^85^ Fragmentomic- and RNA-based assays have also demonstrated potential for detection of early-stage cancers but have not yet been evaluated in prospective studies.^18^^,^^89^

Liquid biopsy may also have a role in the diagnostic process once a cancer has been identified; the tissue specificity of ctDNA methylation patterns is particularly suited to this role. Examples include the use of a pan-cancer enrichment-based methylation assay for diagnosis of cancers of unknown primary cause, a disease where tissue material may be limited or exhausted from initial immunohistochemistry-based testing.^38^ Brain tumors also pose a diagnostic challenge where obtaining tissue via biopsy is technically difficult. CSF sampling via a lumbar puncture offers a complementary source of information and in the context of brain tumors has been shown to have greater sensitivity than plasma for ctDNA detection.^90^^,^^91^ Applying methylation profiling using nanopore sequencing to CSF-derived cfDNA from patients with brain tumors, the correct diagnosis was obtained in 22/110 cases (50/110 with detectable ctDNA by copy-number profiling).^92^

The diagnostic process may be augmented by risk stratification prior to proceeding with invasive investigations. The exosomal RNA-based ExoDx test in prostate cancer can provide information to help patients and clinicians make informed decisions about whether to proceed with a tissue biopsy. This urine test can classify patients with a prostate lesion considering biopsy into “low”- or “high”-risk groups for higher-grade prostate cancer; in a randomized trial, informing patients and clinicians of the score led to a significant reduction in biopsy rates without compromising safety.^93^^,^^94^

### MRD and molecular progression monitoring

In the early disease setting, ctDNA-based assays can be been used to guide adjuvant treatment decisions through monitoring for MRD. The observational GALAXY study of 1,039 patients with resectable stage II–IV CRC identified post-surgical ctDNA-positive status was able to identify which patients would benefit from adjuvant chemotherapy (hazard ratio 6.59, *p* < 0.0001).^95^

In the DYNAMIC study, 455 patients with stage II CRC were randomized in a ratio of 2:1 to ctDNA-guided treatment or standard of care (SOC), with omission of adjuvant chemotherapy in patients with negative ctDNA post-surgery. In the ctDNA-guided arm, rates of adjuvant chemotherapy were 15% compared to 28% in the SOC arm; ctDNA-guided management showed non-inferior 2-year recurrence-free survival (93.5% and 92.4% in the two arms, respectively).^96^ The phase 2 c-TRAK TN study in triple-negative breast cancer used ctDNA to monitor for MRD in 161 patients with randomization to either commence immunotherapy or continue observation if ctDNA became detectable. Although this study demonstrated the technical feasibility of such an approach, there was a disappointingly high rate of radiologically evident metastatic disease at the time of ctDNA detection (72%, 23/32 patients randomized to intervention), highlighting the importance of selecting the right patient cohort and timing of assays if ctDNA monitoring for MRD is to succeed.^97^

### Informing treatment selection

The most established clinical use for cfDNA is as a companion diagnostic for detection of targetable driver mutations in multiple cancer types including lung (EGFR, ALK), ovarian (BRCA1/2), and breast (PIK3CA). FDA-approved tests include both PCR-based and targeted NGS-based assays.^98^ Implementation of these tests in parallel with tissue pathology processing may streamline the identification of actionable mutations that allow access to a targeted therapy or provide a means of testing where insufficient tissue of suitable quality is available. This can facilitate implementation of precision medicine approaches. In the plasmaMatch platform trial, ctDNA testing (by both ddPCR and a targeted panel) was used in a pre-treated cohort of patients with advanced breast cancer.^99^ Of 1,034 patients who underwent testing, 357 had a mutation for which a treatment arm was available, although only 136 patients entered a targeted treatment within the trial and only 2 out of 4 treatment arms achieved their target response rates.

CtDNA-based detection of driver mutations is also being assessed in the context of assigning patients who have exhausted SOC therapies to matched molecularly targeted trial treatments. The TARGET study enrolled patients referred for consideration of early-phase trials who underwent ctDNA and matched tumor molecular profiling.^100^ In the first 100 patients, there was good concordance between tissue- and ctDNA-identified mutations, and 41% had an actionable mutation identified. This has now been expanded to the TARGET National study aiming to recruit 6,000 patients which will use the FoundationOne Liquid CDx ctDNA test to profile 324 cancer-related genes.^101^ Results will be discussed in a national molecular tumor board where available patients will be assigned to a clinical trial of a matched therapy.

### Tracking response and resistance in advanced disease

Increasingly, there is also interest in how the ease of repeat sampling may be exploited to use ctDNA for more precise clinical decision-making throughout a patient’s treatment, such as through tracking of disease status or identifying emerging resistance mechanisms. In the APPLE study in EGFR mutant non-small cell lung cancer (NSCLC), ctDNA detection while on gefitinib of the EGFR T790M mutation (which confers resistance to gefitinib) enabled 17% patients to switch therapy to osimertinib (which T790M mutant NSCLC retains sensitivity to) prior to radiological progression.^102^

Levels of ctDNA reflect disease status and tumor burden while on treatment for a variety of cancers.^103^^,^^104^^,^^105^ In melanoma there is evidence that ctDNA changes may be detectable ahead of radiological or biochemical (LDH) change, giving an early indicator of treatment efficacy or of disease progression.^106^^,^^107^^,^^108^ In the CAcTUS clinical trial, a BRAF V600 ddPCR assay was used to guide a switch from targeted to immune therapy when ctDNA VAF had fallen by ≥80% to provide preliminary data on whether response to an initial run-in of mitogen-activated protein kinase-targeted treatment resulted in improved outcomes for patients with advanced melanoma; final results are awaited.^109^. The upcoming DyNAMic trial (unrelated to DYNAMIC colorectal study) of adaptive vs. continuous targeted therapy in BRAF mutant melanoma will use the same assay to guide timing of adaptive therapy treatment intervals (ISRCTN14643179).^110^ Adaptive therapy involves tailoring treatment windows to an individual’s tumor burden; therefore this strategy requires a biomarker that is a dynamic and accurate marker of tumor activity, hence the choice of a ddPCR ctDNA assay to guide treatment decisions.^111^^,^^112^

### Choice of cfDNA assay for clinical applications

The short turnaround and relative cost-effectiveness of ddPCR testing make it particularly suited to a clinical trial setting where treatment decisions require quantified data to be available in a short time window.^109^ An obvious limitation of ddPCR-based assays for clinical decision making is the requirement for a pre-determined single base pair mutation. Deep targeted sequencing of a panel of genes relevant to the cancer type is an alternative, potentially very sensitive approach. Even deep targeted sequencing can have limited sensitivity when a mutation at the target loci is very low, as may occur in cfDNA. Indeed, a comparison of a targeted panel approach with ddPCR did not show additional benefit for post-operative detection of ctDNA in CRC.^113^

One approach to improve sensitivity of cfDNA mutation calling with targeted sequencing is the integration of variant reads across hundreds to thousands of loci which individually would not meet the threshold of variant calling. This approach was applied in melanoma for longitudinal tracking of cfDNA tumor fraction in advanced melanoma and assessing sensitivity for MRD in a high-risk early-stage cohort of 38 patients from the AVAST-M clinical trial.^114^ In the latter, cfDNA post-surgery was positive in 40% of patients who subsequently relapsed, results which were consistent with a previous study on a cohort from the same trial using ddPCR for cfDNA detection.^20^ These studies relied on a personalized capture panel based on variants detected in tissue samples; while this tumor-informed approach can improve sensitivity, it also increases the cost and challenges of implementation in a clinical setting.

An alternative is to employ WGS which does not require patient-specific panels to be developed and may therefore be amenable to translation to clinical laboratories used to standardized WGS methods. One approach to overcome the limited sensitivity of WGS for variant calling at low VAFs in cfDNA is to exploit the breadth of sequencing through genome-wide variant integration.^62^ This detects and combines tumor mutations across the genome, rather than on the limited number of loci assessed by a targeted panel, resulting in a higher probability of capturing tumor signal. Tumor-specific mutations showed a shorter average fragment size profile, enabling integration of fragment length information into the model to improve performance for tumor fraction detection.^115^ This approach was applied for longitudinal monitoring of treatment response to immune checkpoint inhibitors in melanoma in 37 patients with and early drop in cfDNA tumor fraction corresponding with progression-free survival (PFS) and overall survival (OS) to a greater extent than radiologic disease assessment.^115^ cfRNA is an alternative tumor naive biomarker. An unsupervised analysis of existing melanoma tissue gene expression data identified 4 candidate mRNAs which when quantified in plasma cfRNA using ddPCR were significantly higher in melanoma patients than in healthy donors.^116^ In an expansion cohort of 100 patients, cfRNA level of these mRNAs correlated with stage and tumor burden. Higher baseline mRNA levels were predictive of worse PFS/OS, and increasing levels while on therapy corresponded with radiological disease progression. These results suggest cfRNA may offer a pan-melanoma biomarker for disease monitoring that can be used in those who lack defined hotspot mutations.

While individually the cfNA-based tests are showing promise in specific clinical settings, there has been limited attempt to integrate across multiple ‘omic layers. The sensitivity of cancer detection assays (used for screening or MRD monitoring) where the tumor fraction is expected to be low can be improved by adding additional ‘omic layers.^24^^,^^37^^,^^85^^,^^117^ In one study, integrating fragment-end-motif/length information with copy-number aberrations obtained from shallow WGS improved cancer detection sensitivity compared to either metric alone.^24^ A further example is the integration of cancer-specific methylation signatures and fragment length score obtained from an enrichment-based methylation assay in patients from a pan-cancer phase 2 study of pembrolizumab.^118^ Early changes in these metrics were both independently and jointly predictive of OS. Although the combined score correlated well with a mutation-based score in this study and an early decrease was predictive of OS, its performance was not directly compared to either methylation or fragment length scores alone.^118^

These studies illustrate the potential power of multi-omic liquid biopsies; however, the additional complexity of both performing multi-omic assays and translating the results into a clinically interpretable metric remains a significant barrier to multi-omic tests being translated into clinical practice.

## Tracking tumor biology with multi-omics liquid biopsy

Importantly, the potential of multi-omics liquid biopsy can also be realized in the additional layers of insight it offers into the underlying cancer and cell biology. Understanding the dynamic behavior of tumors and the immune system during therapy requires more frequent and comprehensive sampling than would be feasible from tissue biopsies. The minimally invasive nature of blood cfNA offers an alternative approach for tracking changes in sub-clonal tumor cell populations and the cfNA contributions of different cell populations over time (Figure 2).

### Tracking tumor subclones

The administration of therapeutic agents exerts selective pressures that favor resistant sub-clonal populations, including those carrying previously “neutral” alterations that confer an advantage only in the presence of a therapeutic agent. Clinical resistance may arise through expansion of pre-existing resistant sub-clonal populations.^119^^,^^120^^,^^121^^,^^122^ It is also possible for resistant subclones to arise *de novo*, through acquiring alterations at either the genomic or transcriptomic level.^123^^,^^124^

Multiple studies have leveraged genomic cfDNA analyses to track sub-clonal populations, with clinical correlation with metastatic spread and therapeutic resistance.^120^^,^^122^^,^^124^^,^^125^ In CRC, the emergence of *KRAS* mutant alleles were detectable only after initiation of the EGFR inhibitor cetuximab (which *KRAS* mutations confer resistance to).^126^ In gastrointestinal tumors combining WES of cfDNA, biopsies and autopsy specimens identified resistance mechanisms in 76% patients’ post-progression cfDNA, with more than half exhibiting multiple validated resistance mechanisms, suggesting heterogeneity in acquired resistance.^124^ The TRACERx consortium performed longitudinal tracking of mutations identified in resected NSCLC tissue in cfDNA.^125^ They were able to identify distinct subclone populations and found that subclones identified in metastatic disease were significantly more expanded in pre-operative plasma than non-metastatic subclones.

In melanoma, resistance mutations in genes such as *NRAS* and *PIK3CA* emerge in cfDNA during targeted therapy, although most studies have not specifically examined the parallel existence of sensitive and resistant sub-clonal populations.^123^^,^^127^ Clonal dynamics on therapy were investigated in a patient with *KIT* mutant mucosal melanoma and a mixed clinical response to the cKit inhibitor imatinib: using WES and subsequent targeted sequencing to track ctDNA mutation profiles over time.^121^ Two distinct sub-clonal populations were identified which could be tracked with discordant changes in VAF on multiple lines of therapy.

In a significant proportion of cases, there is a not a clear genetic driver underpinning therapeutic resistance; purely genomic studies would not identify these mechanisms, and in these patients additional ‘omic layers could be valuable.^128^^,^^129^^,^^130^ A recent study in SCLC (small cell lung cancer) utilized reduced representation bisulfite sequencing and identified a switch in methylation profile at the time of progression compared to baseline samples, with a large proportion of patients exhibiting changes in immune-related gene promoter methylation that shifted toward a more inflamed phenotype.^131^

A study in prostate cancer patients integrated cfDNA fragment information with WGS data^122^: one patient exhibited a clonal switch in dominant population at the time of androgen inhibitor resistance where parallel fragment-based inferred gene expression analysis at androgen receptor binding sites demonstrated commensurate loss of downstream androgen receptor signaling. This demonstrates proof of principle that combining genomic and transcriptomic analyses can elucidate clonal evolution with a correlated change in the patient’s developing clinically evident resistance. More multi-omic longitudinal studies are needed to further unravel the complexity of how clinical resistance emerges through changes at different ‘omic levels.

### Tracking cfNAs cell of origin

The contribution of cfNA in the blood by tumor, immune, and normal cells can be affected by many factors that influence aspects of cell biology including cell death and cell turnover; this is therefore a potential avenue to infer information about disease processes, and the cell of origin of cfNA. The role of DNA methylation in cellular differentiation means that the methylation pattern of cell/tissue types can be used to deconvolute the cell type from which cfDNA is derived. This can inform on not just tumor-derived cfDNA but also cfDNA derived from immune cells and from normal tissues as a result of treatment-related toxicity.^14^^,^^17^^,^^132^^,^^133^^,^^134^ This approach was demonstrated in 2015 when methylation markers identified from 14 tissues were applied to cfDNA patients with hepatocellular carcinoma,^14^ reporting the detection of liver-derived cfDNA. A number of methylation “atlases” have been developed based on methylation profiles of individual cell/tissue types.^13^^,^^34^^,^^133^ These data are used to develop algorithms that can deconvolute from a cfDNA sample the contributing proportion of specific cell or tissue types. When applied to cfDNA in patients with cancer, an increase in tissue fraction from the cancer tissue of origin has been observed, with a correlation of increased fraction with cancer stage.^133^ It has been shown in a small number of patients that increases in renal and liver-derived cfDNA post-immunotherapy treatment correlated with biochemical markers, indicating this could also be a marker of treatment-related toxicity.^133^

An alternative approach to cfDNA cellular deconvolution is based on cell-type-specific gene expression profiles. This can either be assessed directly through cfRNA sequencing, or indirectly though fragmentomic analyses that infer gene expression.^67^^,^^135^^,^^136^ A fragment-coverage-based deconvolution method was able to develop cell-type signatures that were predictive of disease and able to correctly differentiate patients with a range of cancer types from controls, including early-stage disease.^65^^,^^136^ A deconvolution algorithm applied to cfRNA from patients with cancer indicated downregulation of CD8+ cytotoxic T cells, B cells, and natural killer cells that was seen in the paired tumor tissue but not paired PBMCs, indicating plasma cfRNA may be a tool to characterize the tumor immune microenvironment.^83^ This work also demonstrated distinct patterns of immune pathway downregulation, e.g., downregulation of cytotoxic T cell and activation and upregulation of the immunosuppressive PD-L1,^83^ indicating the potential complementary power of cfRNA-based studies to explore gene expression.

Individually, these distinct approaches to cfNA cellular deconvolution (methylation, fragmentomic, and RNA-based) offer potential for use in cancer diagnosis, determining tissue of origin and tracking changes in immune/normal tissue contribution throughout therapy that may indicate response or toxicity. What has not yet been shown is whether combining these different, potentially complementary ‘omic deconvolution methods can improve their resolution.

## Integration of multi-omic data from liquid biopsy: Potential and challenges

Liquid biopsy multi-omics approaches generate large amounts of complex data. While integrating these data can offer new biological insights and strengthen development of predictive/prognostic models, careful consideration of how to achieve this is required. The strength of combining data types from cfDNA and other analytes has been demonstrated in a number of studies.^69^^,^^118^^,^^137^^,^^138^ Some of the studies discussed here have performed multiple ‘omic analyses and explored the utility of the different ‘omic layers, e.g., cancer detection or subtype prediction without directly integrating the analysis.^68^^,^^122^ A degree of integration can be achieved by building scores or classifiers based on individual ‘omic layers and then combining the scores or classifier outputs to make an overall prediction.^24^^,^^69^^,^^118^ For example, methylation and fragment features from a single cfDNA workflow were used to build three ‘omic-specific classifiers and a cancer diagnosis prediction was made based on the class with highest average.^69^ Another approach for integrating ‘omic-specific classifier outputs is to use ensemble classifiers.^37^^,^^63^^,^^117^^,^^137^ In one study, regularized logistic regression was used to predict the probability of cancer based on the scores output from four ‘omic-specific classifiers, each based on either methylation or fragmentation features. The classifier outperformed any of the individual scores with higher AUC and lower limit of detection.^37^

An alternative approach is direct integration at the level of the ‘omic data features.^54^^,^^63^^,^^137^^,^^138^ Fragment features are well suited to this; as the size and end-motif profiles of cancer-derived cfDNA are distinct from non-tumor-derived cfDNA, these features can be used to weight genomic features (e.g., copy-number variant [CNV]/single-nucleotide variants [SNVs]) within classifiers for cancer detection based on how likely a fragment is to be tumor/non-tumor derived.^54^^,^^63^ The Lung-CLiP model is an ensemble classifier for early lung cancer detection that integrates SNV and CNV models.^63^ The SNV model incorporates features based on both mutations and fragment length, which improves sensitivity by accounting for the shorter average fragment size of tumor-derived fragments. Another recent study obtained data on five distinct features from combined targeted methylation sequencing and low-pass 0.55× WGBS: methylation changes at target regions, genome-wide methylation, fragment length, copy-number changes, and end motifs from several hundred patients with early-stage cancer and healthy controls.^137^ In addition to using an ensemble classifier for cancer detection, integrating the outputs from individual 'omic-specific classifiers, the authors also tried integrating multi-omic data at the level of the features themselves before feeding the combined data into machine learning algorithms. Interestingly, the ensemble “SPOT-MAS” classifier outperformed all single-feature prediction models whereas the models built directly on combined data were all inferior to both the ensemble classifier and the end-motif single-feature model. This highlights the complexities and nuances of determining the optimal approach to feature integration.

There are a number of challenges when integrating multi-omics data in supervised machine learning models.^139^^,^^140^ Problems with missing data, which can inevitably occur when dealing with clinical samples and complex assays, may be exacerbated if different data types are missing for different individuals or time points across a cohort. The amount of training and validation data available across the different data types can often be a limiting factor, with model overfitting and generalizability of models to new datasets being a potential issue. For ensemble classifier approaches, there is a risk of losing specificity when integrating outputs from multiple ‘omic-specific classifiers, while for approaches that integrate multi-omics features within a single model, there can be the challenge of integrating features of different data types (e.g., continuous and categorical variables) and the number of potential features can differ by orders of magnitude across the different layers. Multi-omic integration in the liquid biopsy setting brings the additional challenge that the tumor-related signal often forms only a small fraction of the total data, which may exacerbate the aforementioned issues.

An issue with any unsupervised integration of heterogeneous multi-omics data is interpretability in terms of deriving the underlying factors that represent sources of variation between samples. Multi-omics factor analysis (MOFA) is one example of a framework that overcomes this issue by inferring hidden “factors” that capture major sources of variation across samples^141^ and then linking these factors back to the specific molecular features that contributed to them. MOFA can disentangle how much each factor is unique to a single modality or shared across multiple modalities, highlighting where different ‘omic layers have either synergized or produced distinct sources of variation. Neither MOFA nor other similar multi-omics approaches^142^ have yet been applied to liquid biopsy, and their utility in this setting remains to be demonstrated.

An important consideration of multi-omics liquid biopsy assays is the possibility of integrating additional clinical, pathological (e.g., biochemistry), and radiological data at multiple time points (Figure 1). This multi-modal data could be used to create a dynamic profile for predicting outcomes (e.g., anticipating therapy resistance or toxicity) which can be refined over time. One such approach to “dynamic risk profiling” involved the creation of a continuous individualized risk index (CIRI) using a Bayesian proportional hazards approach.^143^ These models are disease type specific, combining knowledge of existing clinical/pathological risk factors with serial ctDNA levels. The CIRI-diffuse large B cell lymphoma model produced a personalized prediction of PFS and OS at any given time point throughout the disease course which outperformed existing response assessment tools (e.g., interim positron emission tomography-computed tomography scans). Similarly CIRI models in chronic lymphocytic leukemia and breast cancer performed well.^143^ CIRI selects a small number of existing risk factors to limit the number of predictors and has potential in the longitudinal setting.

## Conclusions

Understanding the complex factors that determine treatment response and the timing and mechanisms of therapy resistance will be key to make further improvements in the outcomes of patients with cancer. Studies of tumor biology based on limited biopsy samples and time points may not be able to fully capture the temporal dynamics of tumor evolution resulting in tumor heterogeneity and differential responses to therapy. Multi-omics liquid biopsy offers a tractable, alternative route to both predict and monitor treatment responses and the acquisition of treatment resistance. Although beyond the scope of this review, cfNA-based liquid biopsies could combine with proteins, tumor-educated platelets, and lipids, alongside integration with radiomic, pathological, and clinical data. Current methods for ‘omic analyses face limitations in terms of clinical applicability due to cost, specialist sample and data handling requirements, and turnaround time. Although novel approaches have increased sensitivity of ctDNA-based detection, there is still a limit to this that may be a barrier for its use in monitoring MRD, as the time window between molecularly and clinically detectable relapse must be sufficient to be clinically meaningful. One recently proposed technique to enhance cfDNA assay sensitivity is the use of *in vivo* “priming” agents which transiently reduce the rate of cfDNA clearance; these agents are, however, some way from deployment in humans.^144^

For assays to be implemented in clinical laboratories, a range of variables at both the pre-analytical (e.g., sample collection tube, storage, and processing) and analytical stages require standardization and technical validation.^145^^,^^146^ Academic institutions where a lot of these tests are initially developed can have varied practices and access to accredited laboratories (ISO 15189/FDA accredited) for analytical validation can be costly/require commercial investment. A further challenge if ‘omic liquid biopsies are clinically utilized as predictive or prognostic biomarkers is the necessity for robust, validated bioinformatics pipelines that can account for pre-analytical variation and that produce an output that is clinically meaningful and understandable for clinicians and their patients when making treatment decisions. The advantages of adding ‘omic layers that provide additional information will need to be balanced against the requirements for outputs that can be straightforwardly and consistently applied to clinical decision-making and assessed within the structure and regulatory requirements of a prospective clinical trial. Developing regulatory roadmaps for the robust assay validation required for clinical implementation represents another challenge for these complex multi-omic liquid biopsies. Some of the more complex techniques and approaches discussed may be more relevant for understanding the biological underpinnings of tumor biology and emergence of resistance than being directly applied in the clinic. Finally, for any assay to be used in the clinic, it must first demonstrate that it can improve outcomes for patients preferably within a prospective phase 3 clinical trial.

While therapeutic advances over the past decade have transformed cancer outcomes, clinicians, researchers and patients are searching for ways to improve outcomes for those who do not respond to therapy or who develop resistance, as well as avoiding unnecessary overtreatment and associated toxicities. The breadth of data that ‘omic liquid biopsies can provide at multiple time points throughout a patient’s journey with cancer can be used to aid diagnosis, refine treatment decisions, and understand the mechanisms by which resistance develops in order to improve patient outcomes in the future.

## Acknowledgments

The work was funded by 10.13039/501100000289Cancer Research UK (CRUK) via core funding to the 10.13039/501100000289CRUK National Biomarker Centre and supported by the Manchester Experimental Cancer Medicines Centre, the 10.13039/501100020252CRUK Lung Cancer Centre of Excellence, and the 10.13039/100014653NIHR Manchester Biomedical Research Centre. A.T. is funded by the 10.13039/501100000289CRUK Manchester Centre (C147/A25254) and their Clinical Academic Training Award (C19941/A28707).

## Declaration of interests

F.M. is a co-inventor on multiple patents related to cfDNA analysis and has consulted for Roche Dx. A.C., S.M.H., D.G.R., and C.D. are co-inventors on a patent relating to cfDNA analysis. R.J.L. has received a speaker fee from Pierre Fabre and research funding from Bristol Myers Squibb, AstraZeneca, and Pierre Fabre. C.D. has received research funding/educational research grants since 2020 from the following: AstraZeneca, Amgen, Carrick Therapeutics, Merck AG, Bayer, Boehringer Ingelheim, BMS, Novartis, Celgene, Epigene Therapeutics Inc, and Menarini. C.D. has received honoraria for consultancy and/or advisory boards from Merck, AstraZeneca, GRAIL, and Boehringer Ingelheim.
